# Matrix Metalloproteinase 9 Secreted by Hypoxia Cardiac Fibroblasts Triggers Cardiac Stem Cell Migration *In Vitro*

**DOI:** 10.1155/2015/836390

**Published:** 2015-02-12

**Authors:** Qing Gao, Maojuan Guo, Wenyun Zeng, Yijing Wang, Lin Yang, Xiaoli Pang, Huhu Li, Yanrong Suo, Xijuan Jiang, Chunquan Yu

**Affiliations:** ^1^School of Integrative Medicine, Tianjin University of Traditional Chinese Medicine, Tianjin 300193, China; ^2^Periodical Newsroom, Tianjin University of Traditional Chinese Medicine, Tianjin, China

## Abstract

Cessation of blood supply due to myocardial infarction (MI) leads to complicated pathological alteration in the affected regions. Cardiac stem cells (CSCs) migration plays a major role in promoting recovery of cardiac function and protecting cardiomyocytes in post-MI remodeling. Despite being the most abundant cell type in the mammalian heart, cardiac fibroblasts (CFs) were underestimated in the mechanism of CSCs migration. Our objective in this study is therefore to investigate the migration related factors secreted by hypoxia CFs *in vitro* and the degree that they contribute to CSCs migration. We found that supernatant from hypoxia induced CFs could accelerate CSCs migration. Four migration-related cytokines were reported upregulated both in mRNA and protein levels. Upon adding antagonists of these cytokines, the number of migration cells significantly declined. When the cocktail antagonists of all above four cytokines were added, the migration cells number reduced to the minimum level. Besides, MMP-9 had an important effect on triggering CSCs migration. As shown in our results, MMP-9 induced CSCs migration and the underlying mechanism might involve TNF-*α* signaling which induced VEGF and MMP-9 expression.

## 1. Introduction

Ischemic heart disease leads to cardiomyocytes necrosis or apoptosis and eventually cardiac insufficiency and myocardial infarction (MI). Resident cardiac stem cells (CSCs) can repair damaged myocardium and improve myocardial function in both human and animal [1]. In recent years, various studies have shown that CSCs are capable of self-renewing and proliferating as well as differencing into cardiomyocytes, endothelial, and smooth muscle cells [2]. Besides, CSCs are able to migrate into the site of heart injury and participate in restoring myocardium that was damaged. So the cardioprotective effect of CSCs relies not only on its proliferation, but also on its ability to migrate towards injury within the heart [3]. Signaling factors and pathways that were shown to participate in CSCs migration process include hypoxia-inducible factor-1*α* (HIF-1*α*), stromal cell-derived factor-1 (SDF-1)/CXCR4, stem cell factor (SCF)/c-kit, hepatocyte growth factor (HGF)/c-met, insulin-like growth factor-1 (IGF-1)/IGF-1R, erythropoietin (EPO)/EPOR, EphrinA1/EphA1 Receptor, and so on [4–6]. However, the mechanism that how chemotactic factors attract CSCs migration has not yet been fully understood.

The majority of mesenchymal cells in the heart are fibroblasts, which present stronger resistance against ischemic and anoxia injury than cardiomyocytes do. Cardiac fibroblasts (CFs) are well recognized as active participants in cardiac remodeling after MI. Its primary effect is to produce proteins including interstitial collagens, proteoglycans, glycoproteins, matrikines, and metalloproteinases, all of which contribute to extracellular matrix (ECM). Therefore, the heart injury can be repaired by fibers [7]. In response to inflammatory mediators, resident CFs and other cell lineages are stimulated to differentiate into myofibroblasts, which results in abundant ECM synthesis and deposition [8]. Taking into account the important role of CFs in tissue repair, we are interested in testing the effect of CFs in triggering CSCs migration, especially the possible mechanism under hypoxia condition.

## 2. Material and Methods

### 2.1. Statement of Ethics

The experimental procedures on animals were in strict accordance with Provisions and General Recommendation of Chinese Experimental Animals Administration Legislation and was approved by the Committee on Ethics of Animal Experiments of the Tianjin University of Traditional Chinese Medicine (Permit number SCXK2009-003). All surgeries were performed under sodium pentobarbital anesthesia with minimize animal suffering.

### 2.2. Isolation, Culture, and Purification of Mouse Cardiac Fibroblasts

CFs were isolated and cultured as described previously. In brief, neonatal mice (1-2 days old, *n* = 20) were sterilized in 75% alcohol for 1 min after euthanized with CO_2_. Pericardium/epicardium and endocardium were discarded after chest opening while the myocardial tissues (auricles and ventricles) were excised and washed in ice-cold D-Hank's (Gibco, USA) solution and then cut into 1 mm^3^ pieces. Tissues were then digested with 0.08% pancreatic enzyme (Hyclone, USA) and 0.1% collagenase II (Invitrogen, USA) at 37°C for 8 min with oscillation. The supernatant was collected and filtered through a 400 *μ*m nylon mesh filter and then centrifuged at 1000 g for 8 min. Cells harvested from supernatants were then seeded in 25 cm^2^ flask and cultured with DMEM/F12 (Gibco, USA) contained 10% FBS (Hyclone, USA) and 1% Penicillin-Streptomycin (Hyclone, USA) at 37°C with 5% CO_2_ in a humid incubator. The medium was exchanged every other day. CFs were identified with antivimentin (Santa Cruz, catalog number sc-7558, 1 : 200) using immunofluorescence.

### 2.3. Cardiac Fibroblasts Hypoxia Treatment

CFs were incubated for 0 h, 12 h, 24 h, and 48 h in hypoxia incubator (5% CO_2_ + 94% N_2_ + 1% O_2_), until cells reached at least 90% confluence in a 6-well plate. Subsequently, supernatants were collected for chemotaxis assay and ELISA, while cells were pertained for qPCR.

### 2.4. Cell Vitality Assay

Cell vitality assay was measured with the cell counting kit-8 (CCK-8; DOJINDO, Kumamoto, Japan) at 0 h, 12 h, 24 h, and 48h under hypoxia following the manufacturer's instructions.

### 2.5. Cell Chemotaxis Assay

Cell migration assay was performed with transwell inserts (Corning Costar Corporation, 8 mm pore filter, 24-well cell clusters). The mouse CSCs were placed into the transwell insert with 100 *μ*L culture medium (DF12 + 10% FBS). Supernatants (600 *μ*L) of CFs from each period of hypoxia incubation were added into the lower chambers as chemotaxin. After 12 h, the cells on the upper side of the membrane were removed. The cells on the lower surface were air-dried, fixed, and stained with crystal violet (Solarbio, China). Cells were counted at high magnification under a microscope (Leica DM3000B, German).

### 2.6. Quantitative Reverse Transcriptase Polymerase Chain Reaction (qPCR)

Total RNA was extracted from cultured CFs using Trizol (Ambion, USA). RNA concentration was determined by microspectrophotometer. Reverse transcription of mRNA into cDNA was performed according to the manufacturer's protocol (Tiangen, China). mRNA expression levels were quantified using a qPCR system (Fast 7500, ABI). qPCR reactions were performed with SYBR Green kit (Tiangen, China), using 1 *μ*L of cDNA as a template in each 25 *μ*L reaction mixture. The PCR protocol was as follows: an initial 15 min denaturation at 95°C followed by 40 cycles involving 10 s denaturation at 95°C, 30 s annealing at 60°C, and 30 s extension at 72°C. Transcription levels of migration-related factors, including vascular endothelial growth factor (VEGF), tumor necrosis factor-*α* (TNF-*α*), matrix metalloproteinase-9 (MMP-9), matrix metalloproteinase-2 (MMP-2), stem cell factor (SCF), tissue inhibitor of metalloproteinase-1 (TIMP1), epidermal growth factor (EGF), stromal cell-derived factor 1 (SDF-1), basic fibroblast growth factor (bFGF), granulocyte colony-stimulating factor (G-CSF), hepatocyte growth factor (HGF), hypoxia inducible factor-1*α* (HIF-1*α*), and monocyte chemoattractant protein-1 (MCP-1) were tested. Their primer sequences are listed in Table 1. The quantitative reactions were conducted according to the manufacture's instruction (Agilent, USA).

### 2.7. Enzyme-Linked Immune Sorbent Assay (ELISA)

The supernatants from hypoxia cultured CFs were used for ELISA assay. Based on qPCR results, the upregulated factors including vascular endothelial growth factor (VEGF), tumor necrosis factor-*α* (TNF-*α*), matrix metalloproteinase-9 (MMP-9), and matrix metalloproteinase-2 (MMP-2) were tested using ELISA kit (USCN, USA).

### 2.8. Chemotaxis Blockage Assay

Chemotaxis blockage assays were performed using Costar Transwell inserts after specific chemokine antagonists were added in the supernatants. In brief, the cytokine antagonists used to block the activity of specific growth factors were EYE001 (VEGF antagonist, pegaptanib sodium, Eyetech Pharmaceuticals), etanercept (TNF-*α* antagonist, Wyeth Pharmaceutical, Japan), ab142180 (MMP-9 inhibitor, Abcam, USA), and SB-3CT (MMP-9/2 inhibitor, Abcam, USA). Each antagonist was dissolved in PBS and added to supernatant of CFs, which were treated with hypoxia for 24 h to a final concentration of 1 *μ*g/mL. Meanwhile, a cocktail of these four antagonists was added in the same final concentration. Supernatant of CFs for hypoxia 0 h was used as control group and added with normal PBS. All groups were incubated for 2 h before the chemotaxis assay was performed to allow interaction of these factors and their antagonists. After a further incubation of 12 h the migration cells number was counted in chemotaxis assay.

## 3. Results

### 3.1. The Identity and Vitality of Cultured CFs under Hypoxia Condition

**Figure 1:**
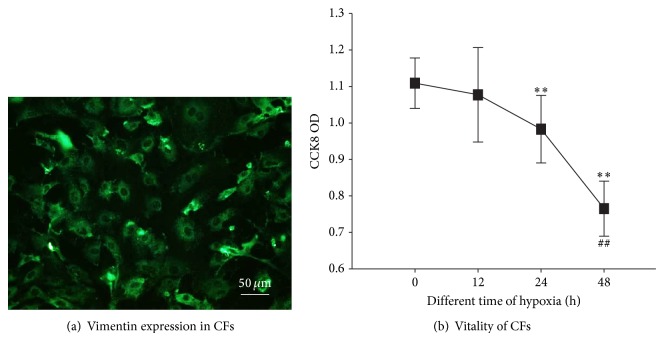
Vimentin staining and vitality of CFs under hypoxia. (a) CFs with vimentin marker was identified by green immunofluorescence. (b) Cell vitality curve depicted using time points of 0 h, 12 h, 24 h, and 48 h under hypoxia. Cell vitality dropped in a timely dependent manner. Results are shown as the mean ± SD values. Experiments were repeated three times. ∗∗ indicates *P* < 0.01 compared to the 0 h group. ## indicates *P* < 0.01 compared to the 24 h group.

**Figure 2:**
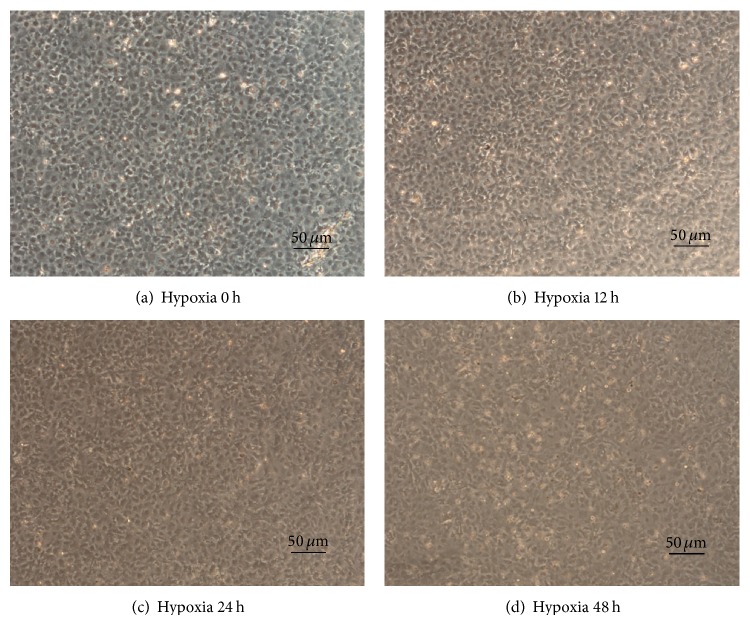
CFs morphological changes under hypoxia. (a) shows the control when CFs were to be incubated under hypoxia. (b) (12 h), (c) (24 h), and (d) (48 h) demonstrate that CFs were incubated by different length under hypoxia. CFs became less stereoscopic in the hypoxic condition.

Overwhelming majority of cultured cells are positive for vimentin (Figure 1(a)), which was the marker of CFs. Cell vitality declined under hypoxia condition in a timely dependent manner (Figure 1(b)). Compared to CFs under normoxia (Figure 2(a)), the CFs in the hypoxic condition became less stereoscopic (Figures 2(b), 2(c), and 2(d)).

### 3.2. Hypoxia-Induced CFs Increased CSCs Migration in a Time-Dependent Manner

**Figure 3:**
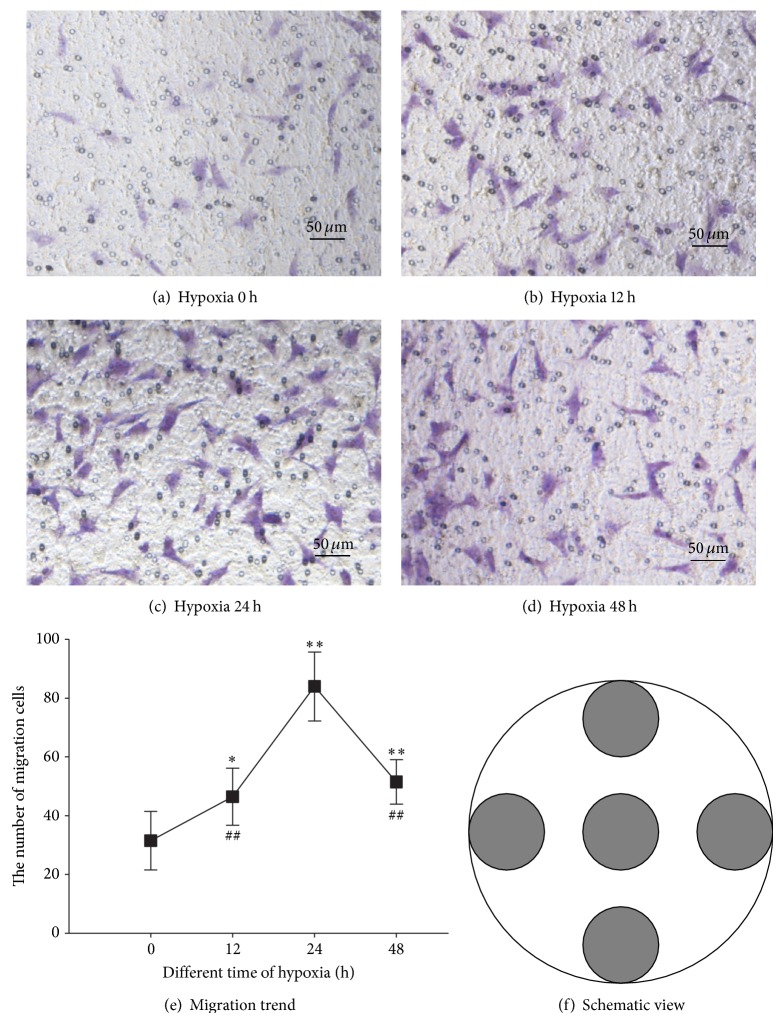
Supernatant of hypoxia induced CFs promoted CSCs migration. (a) shows CFs were exposed as control under normoxia while (b) (12 h), (c) (24 h), and (d) (48 h) demonstrate that CFs were cultured under different time period of hypoxia. (e) reveals the migration curve under different periods of hypoxia that peaks at 24 h. (f) denotes CSCs migration accounts in the well are counted according to the fixed location. ∗ indicates *P* < 0.05 compared to the 0 h group. ∗∗ indicates *P* < 0.01 compared to the 0 h group. ## indicates *P* < 0.01 compared to the 24 h group.

Encountering myocardial infarction, affected cardiac cells especially CFs suffered from hypoxia crisis. To confirm whether CFs under hypoxia for different time periods were capable of directing the migration of CSCs* in vitro*, we used Costar Transwell inserts, where CSCs and conditioned medium from hypoxia-induced CFs were separated by a microporous membrane that allows cell passage. Our data showed conditioned medium from hypoxia-induced CFs attracted CSCs migration in a time-dependent manner (Figure 3). The chemotaxis of CFs exposed to hypoxia peaks at 24 hours. Therefore, we raise the hypothesis that cytokines in the supernatants might contribute to the CSCs migration. Then mRNA transcription and protein expression levels of migration-related cytokines in the supernatants of cultured CFs were examined.

### 3.3. Hypoxia Upregulated Transcription Levels of Some Migration Related Factors

**Figure 4:**
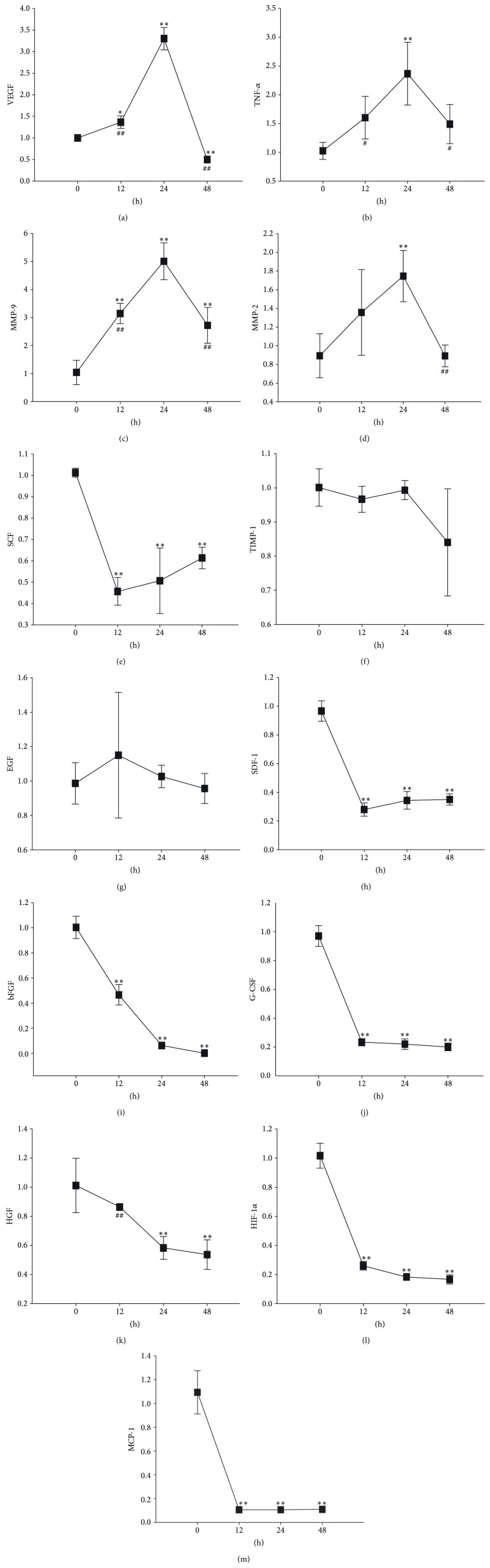
Seven migration related factors induced by supernatant of hypoxia CFs culture were upregulated in mRNA transcription level. VEGF (a), TNF-*α* (b), MMP-9 (c), MMP-2 (d), SCF (e), TIMP-1 (f), EGF (g), SDF-1 (h), bFGF (i), G-CSF (j), HGF (k), HIF-1*α* (l), and MCP-1 (m) mRNA transcription levels in supernatants of CFs under different period of hypoxia. mRNA levels of VEGF, TNF-*α*, MMP-9, and MMP-2 mRNA peaked at 24 h, while mRNA levels of SCF, SDF-1, bFGF, G-CSF, HGF, HIF-1*α*, and MCP-1 declined in a timely dependent manner with the period of hypoxia treatment. Lastly, mRNA levels of EGF and TIMP-1 showed no change with hypoxia treatment at all. Results are shown as the mean ± SD values. Experiments were repeated three times. ∗ indicates *P* < 0.05 compared to the 0 h group. ∗∗ indicates *P* < 0.01 compared to the 0 h group. # indicates *P* < 0.05 compared to the 24 h group. ## indicates *P* < 0.01 compared to the 24 h group.

To understand the CSCs migration under hypoxia condition in detail, we used qPCR to examine mRNA transcription levels of migration related factors in the supernatant of cultured CFs under hypoxia for 0 h, 12 h, 24 h, and 48 h, respectively. Literature search indicates that migration-related factors include VEGF, TNF-*α*, MMP-9, MMP-2, SCF, TIMP-1, EGF, bFGF, SDF-1, G-CSF, HGF, HIF-1*α*, and MCP-1. Our data revealed that VEGF, TNF-*α*, MMP-9, and MMP-2 mRNA transcription levels were upregulated and peaked at 24 h under hypoxia while transcription levels of SCF, SDF-1, bFGF, G-CSF, HGF, HIF-1*α*, and MCP-1 were downregulated. Meanwhile, transcription levels of EGF and TIMP-1 almost had no significant difference under hypoxia compared to normoxia (Figure 4).

### 3.4. Hypoxia Upregulated Protein Expression Levels of Some Migration-Related Factors

**Figure 5:**
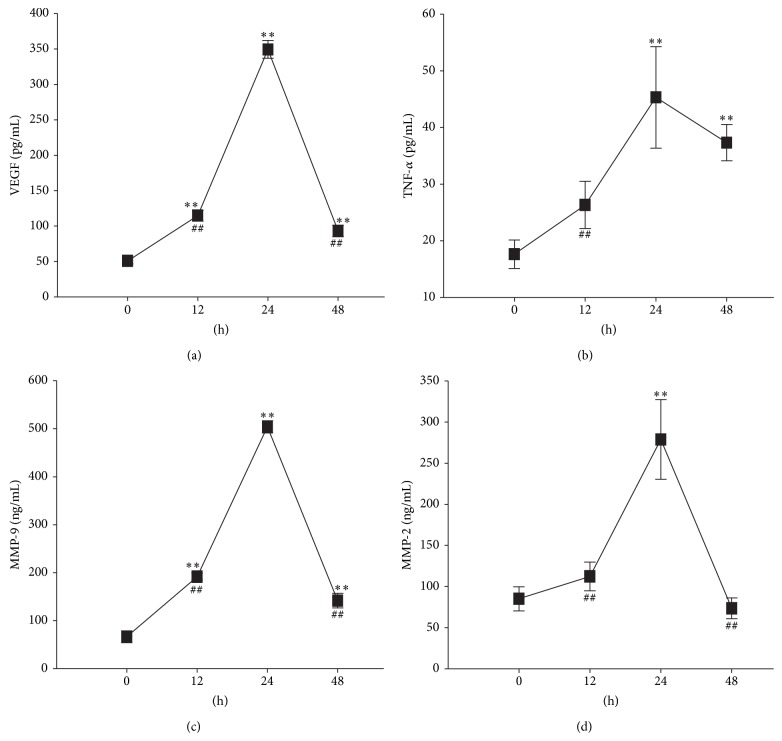
Protein levels of the four migration related factors whose mRNA level was also upregulated. The protein levels of those factors that were upregulated in mRNA level under hypoxia were also increased and peaked at 24 h as shown in (a) (VEGF), (b) (TNF-*α*), (c) (MMP-9), and (d) (MMP-2). Results are shown as the mean ± SD values. Experiments were repeated three times. ∗ indicates *P* < 0.05 indicates compared to the 0 h group. ∗∗ indicates *P* < 0.05 indicates compared to the 0 h group. # indicates *P* < 0.05 compared to the 24 h group. ## indicates *P* < 0.01 compared to the 24 h group.

Although the transcription levels of VEGF, TNF-*α*, MMP-9, and MMP-2 were upregulated in mionectic CFs, their protein expression levels were unclear. So we employed ELISA to examine these factors which revealed that protein expression levels of VEGF, TNF-*α*, MMP-9, and MMP-2 in supernatants were upregulated as their corresponding mRNA did (Figure 5).

### 3.5. Role of MMP-9 in Chemotactic Attraction

**Figure 6:**
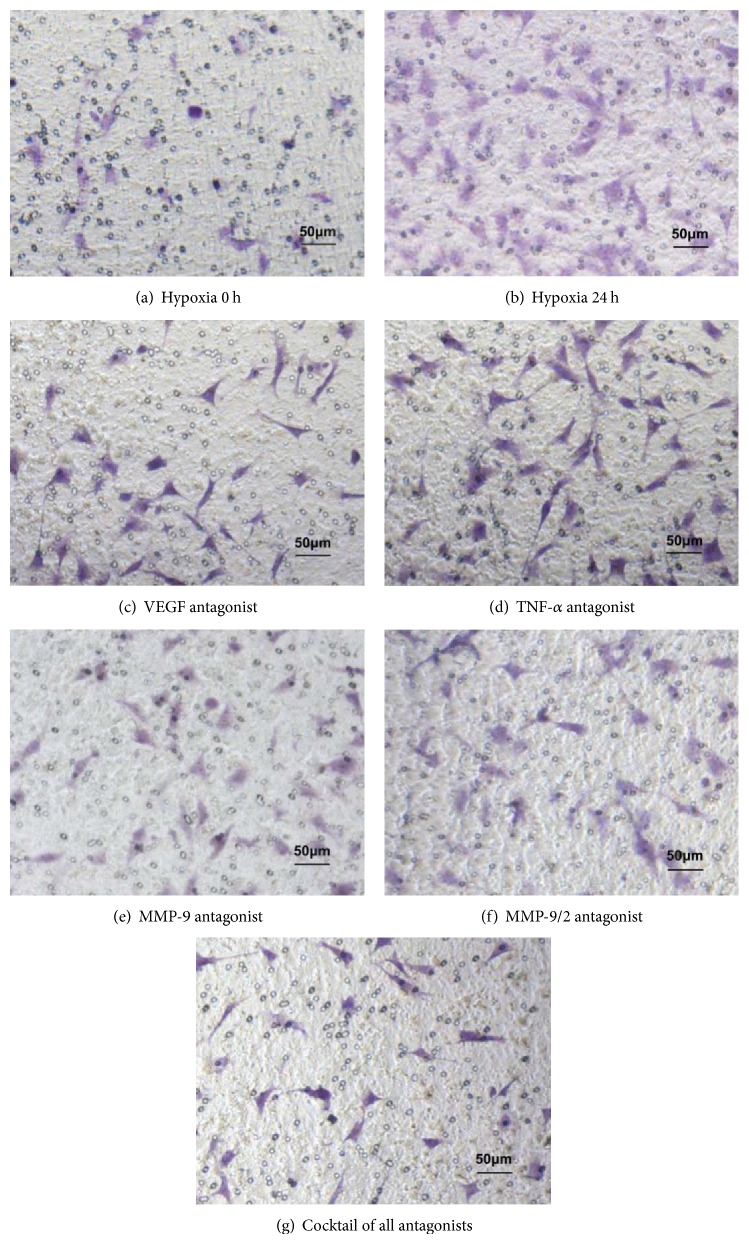
CSCs migration assay with cytokine antagonists. (a) shows CSCs migration induced by supernatant of CFs culture just before being incubated under hypoxia (0 h group). Then, CSCs migration induced by supernatant of CFs culture incubating under hypoxia for 24 h was shown in (b). Antagonists for VEGF (c), TNF-*α* (d), MMP-9 (e), MMP-2 (f), and cocktail of all four factors (g) were added to the cells that were induced by CFs culture under hypoxia for 24 h. When all the antagonists were added, the CSCs migration number declined to a minimum level.

**Figure 7:**
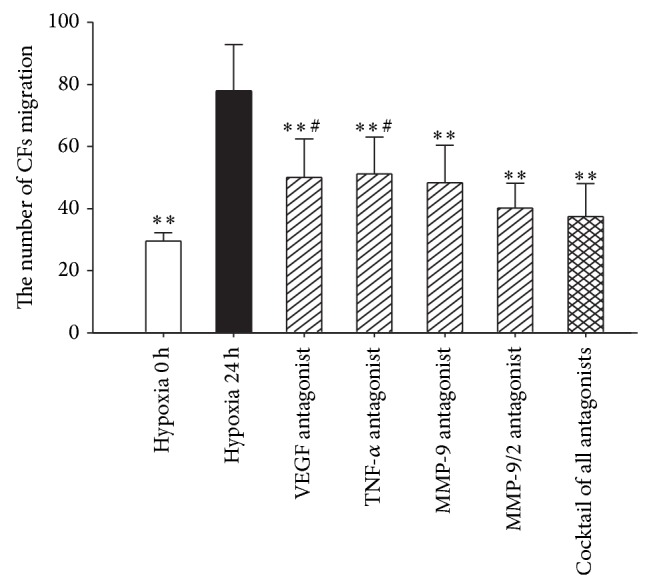
CSCs migration number after adding antagonists. Each of these factors partly participated in the migration process. Compared to the group that was added with cocktail antagonist against all four factors, the number of migration cells in the group that was added with MMP-9 antagonist and MMP-9/2 antagonist shows no significant difference. Results were shown as the mean ± SD values. Experiments were repeated three times. ∗ indicates *P* < 0.05 compared to the hypoxia 24 h group. ∗∗ indicates *P* < 0.01 compared to the hypoxia 24 h group. # indicates *P* < 0.05 compared to the group added with the cocktail of all antagonists. ## indicates *P* < 0.01 compared to the group added with the cocktail of all antagonists.

As shown in Figures 6 and 7, the number of migration cells in each antagonist group under hypoxia for 24 h significantly declined compared to the hypoxia 24 group without antagonists. When the cocktail of all antagonists was added at the same time, the migration cells number reduced to a minimum level. Due to the unavailability of MMP-2 inhibitor in market, we replace MMP-9/2 antagonist which shows no significant difference with MMP-9 used alone. Both the MMP-9 antagonist group and the MMP-9/2 antagonists group had no significant difference compared to the cocktail of all antagonists group. Combined with the qPCR results, in which MMP-9 demonstrates the most significant upward trend, we deduce that MMP-9 acts as an essential role in CSCs migration.

## 4. Discussion

Despite CFs comprise approximately two-thirds of total cells number in the heart, its role in cardiac development, physiology, and disease pathogenesis was under estimated [9]. CFs play a critical role in orchestrating injury response [10]. Moreover, the expression of cytokines from CFs is markedly altered in pathological conditions. CFs are the main source of proinflammatory factors, which enhance fibroblasts migration and increase secretion of MMPs after MI [11]. However, fiber accumulation leads to cardiac fibrosis and cardiac insufficiency. So how CFs behave under pathological conditions remains to be elucidated.

In recent years, cardiac stem cells (CSCs) show protective role in heart disease [12–14]. Most of CSCs are located in the atria, apex, and base-mid region of the ventricle [15]. Studies shows CSCs are self-renewing, clonogenic, and multipotent, which could differentiate into three major cardiac lineages: cardiomyocytes, vascular smooth muscle cells, and endothelial cells [16]. Bolli et al. infused autologous CSCs to patients with ischemic cardiomyopathy and found that CSCs are effective in improving LV systolic function and reducing infarct size [17] which was consistent with several other reports [18–21]. Furthermore, CSCs can be attracted by cytokines especially growth factors to migrate into the infarct area [22–24]. But the source of these factors and their exact role remains unclear.

In our present study, we focused on the paracrine effects of CFs under hypoxia and those cytokines secreted by CFs contribute to CSCs migration. Blockage of coronary arteries leads cessation of blood flow to myocardium and finally results in reduced supply of oxygen and then necrosis of the affected regions.* In vitro* cell culture mimics the anoxic condition by incubating CFs in hypoxia incubator. As expected, our data showed a time-dependent manner of CSCs migration promoted by hypoxia-induced CFs. The maximal chemotaxis was observed at 24 h under hypoxia after CFs were cultured.

Previous studies reported that chemokines, such as VEGF, TNF-*α*, MMP-9, and MMP-2 trigger CSCs migration* in vivo* and* in vitro*. However, the expression levels of these chemokines in hypoxia-induced CFs and their contribution to CSCs migration are still unclear. In the present study, we confirmed the upregulation of VEGF, TNF-*α*, MMP-9, and MMP-2 transcription and expressions by qPCR and ELISA, respectively, upon addition of secretions from hypoxia-induced CFs. In addition, expression levels of VEGF, TNF-*α*, MMP-9, and MMP-2 peaked concurrently with maximum CSCs migration under hypoxia 24 h. It was insinuated that these cytokines might play a momentous role in CSCs migration.

To further characterize these factors, we added their corresponding inhibitors before migration assay. When VEGF, TNF-*α*, MMP-9, and MMP-2 were inhibited independently, the number of CSCs migration was declined to various extends. When these factors were inhibited together, CSCs migration rate was reduced to the lowest level among all groups. It is exciting to find that MMP-9 antagonist showed similar result as cocktail of all four antagonists. It indicated that MMP-9 plays a key role in triggering CSCs migration.

MMP-9 is the primary gelatinase that is able to degrade collagen IV, which is one of the major components of basement membrane secreted by keratinocytes. Increasing evidences suggest that MMPs play a critical role in vascular formation and remodeling through degrading vascular basement membrane and ECM as well as modifying angiogenic growth factors and cytokines [25]. Heissig et al. concluded that VEGF stimulates the release of pro-MMP-9 and induces migration of human CD34 progenitor and stem cells [26]. VEGF has been recognized as a chemotactic factor over years. Grunewald et al. found that VEGF mobilizes homing of bone marrow-derived circulating cells through SDF-1/CXCR4 pathway [27, 28]. Tang et al. reported VEGF promoted cardiac stem cells migration via PI3K/Akt pathway [29]. Another result also demonstrated that TNF-*α* stimulates MMP-9 expression by activating p38 MAPK signaling pathway [30]. Additionally, tumor-cell-derived TNF-*α* directly accelerates MMP-9 expression in fibroblasts [31]. TNF-*α* elevates the latent form of MMP-9 in a dose dependent manner. Therefore, we speculate that TNF-*α* facilitates VEGF and MMP-9 expression besides its role in cross-talking between CFs under hypoxia condition.

Chemokines, such as SCF, SDF, G-CSF, EGF, HGF, HIF-1*α*, and MCP-1, were also reported to activate CSCs migration* in vivo* and* in vitro* [32–37]. However, our consequences propose that these migration factors were not secreted by hypoxia induced CFs, but may be secreted by other cells after MI, such as cardiomyocytes, macrophages, and inflammatory cells. Among these factors, HIF-1*α* draws our attention since it is a pivotal regulator of the hypoxic response following myocardial infarction [38]. However, hypoxia induced CFs did not provoke HIF-1*α* expression in the present result, which is similar to the research of Nikami et al. [39]; hence the function of HIF-*α* in CFs needs further investigation.

In conclusion, we investigated the possible role of hypoxia-induced CFs on CSCs migration. Our data exhibit the expression of CFs-associated factors including VEGF, TNF-*α*, MMP-9, MMP-2, SCF, TIMP-1, EGF, SDF-1, G-CSF, HGF, HIF-1*α*, and MCP-1 during various periods of hypoxia damage* in vitro.* It also clearly showed that hypoxia-induced CFs could promote CSCs migration via TNF-*α*, MMP-9, and VEGF, and MMP-9 played the dominant role. The chemokines were proved to be effective for mediating CSCs migration in the present study and might be used as potentially therapeutic molecular targets in ischemia cardiac disease using cell transplantation.

## Figures and Tables

**Table 1:** Primer sequences in qPCR assay.

Primer	Sequence (5′ to 3′)	Product size (bp)	Gene accession number
TIMP-1-F	CCTTTGCATCTCTGGCATCT	141	NM_001044384
TIMP-1-R	GGGAACCCATGAATTTAGCC		
MMP-9-F	TCCGTGTCCTGTAAATCTGCT	152	NM_013599
MMP-9-R	GACCTGAACCATAACGCACA		
MMP-2-F	TTCAACGGTCGGGAATACAG	103	NM_008610
MMP-2-R	AGCCATACTTGCCATCCTTC		
TNF-*α*-F	CACCACCATCAAGGACTCAA	102	NM_001278601
TNF-*α*-R	GAGACAGAGGCAACCTGACC		
bFGF-F	AGGAAGATGGACGGCTGCT	138	NM_008006
bFGF-R	GCCCAGTTCGTTTCAGTGC		
HIF-1*α*-F	TGAACATCAAGTCAGCAACG	129	NM_010431
HIF-1*α*-R	CACAAATCAGCACCAAGCAC		
SDF-1-F	TCTTAGGAGGCACAGCAAGC	101	NM_001012477
SDF-1-R	CAGGCATTTCCATGAGGACT		
G-CSF-F	TGCACTATGGTCAGGACGAG	108	NM_009971
G-CSF-R	CTCACTTGCTCCAGGGACTT		
EGF-F	GCTCTTCTGGGTTCAGGACA	112	NM_010113
EGF-R	AGACAAACTGTGCCGTGCTT		
HGF-F	TGCTCCTCCCTTCCCTACTC	109	NM_001289458
HGF-R	CGGGCTGAAAGAATCAAAGC		
VEGF-F	CTGCTGTGGACTTGTGTTGG	106	NM_001025250
VEGF-R	AAAGGACTTCGGCCTCTCTC		
MCP-1-F	CCCACTCACCTGCTGCTACT	101	NM_011333
MCP-1-R	CAGCTTCTTTGGGACACCTG		
GAPDH-F	GGAGCCAAACGGGTCATCATCTC	121	NM_008084
GAPDH-R	AGTGGGAGTTGCTGTTGAAGTCGC		
